# 3-{[Bis(pyridin-2-ylmeth­yl)amino]­meth­yl}-2-hy­droxy-5-methyl­benz­aldehyde

**DOI:** 10.1107/S1600536812019940

**Published:** 2012-05-12

**Authors:** Ruo-Xu Wang, Da-Zhi Gao, Fan Ye, Yan-Fei Wu, Dun-Ru Zhu

**Affiliations:** a College of Chemistry and Chemical Engineering, State Key Laboratory of Materials-Oriented Chemical Engineering, Nanjing University of Technology, Nanjing 210009, People’s Republic of China

## Abstract

In the title compound, C_21_H_21_N_3_O_2_, the pyridine rings and the benzene ring lie in a propeller arrangement around the central tertiary amine N atom. The dihedral angles formed by the benzene ring with the pyridine rings are 61.0 (3) and 49.6 (3)°, while the dihedral angle between the pyridine rings is 69.7 (3)°. The mol­ecular conformation is stabilized by intramolecular bifurcated O—H⋯N hydrogen bonds. In the crystal, inversion dimers are formed *via* pairs of C—H⋯N hydrogen bonds.

## Related literature

For general background to unsymmetric phenolate compounds, see: Lambert *et al.* (1997 ▶); Dubois, Xiang *et al.* (2003 ▶); Dubois, Caspar *et al.* (2003 ▶); Carlsson *et al.* (2004 ▶). For the syntheses and structures of related compounds, see: Chirakul *et al.* (2000 ▶); Abe *et al.* (2006 ▶); Bortoluzzi *et al.* (2007 ▶); Koval, Huisman, Stassen, Gamez, Lutz, Spek & Reedijk (2004 ▶); Koval, Huisman, Stassen, Gamez, Lutz, Spek, Pursche *et al.* (2004 ▶); Koval *et al.* (2007 ▶); Zhu *et al.* (2007 ▶). For the synthesis of the title compound, see: Lambert *et al.* (1997 ▶); Koval *et al.* (2003 ▶).
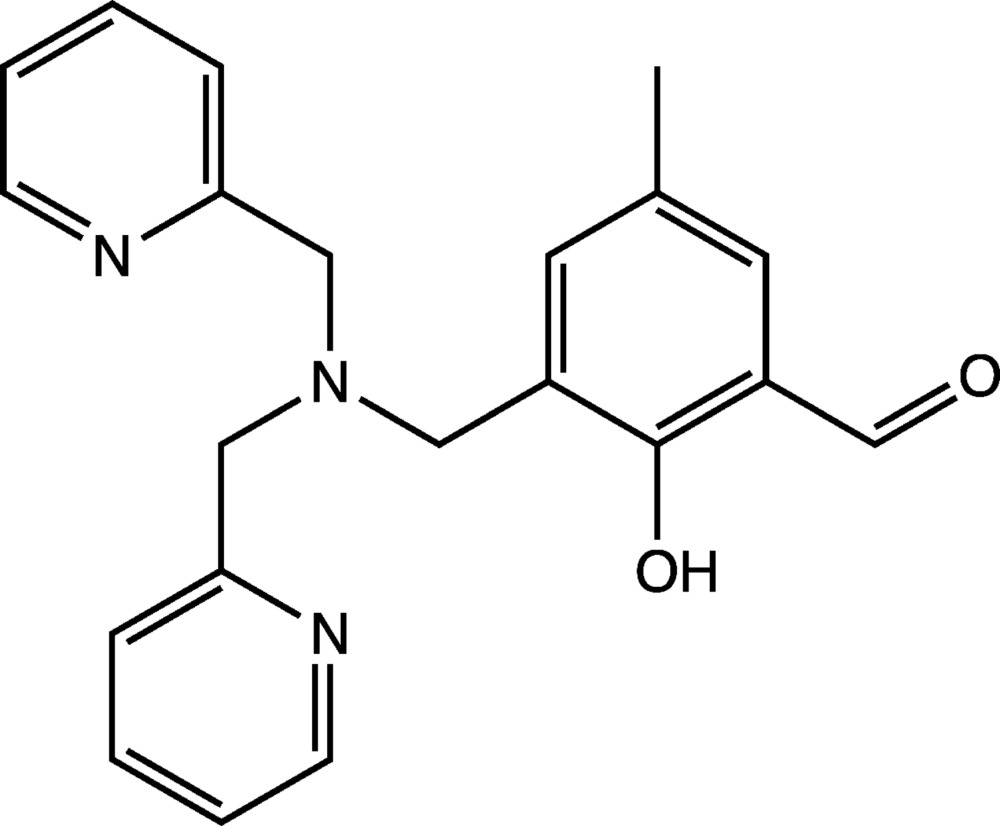


## Experimental

### 

#### Crystal data

C_21_H_21_N_3_O_2_
*M* *_r_* = 347.41
Triclinic, 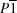

*a* = 8.479 (3) Å
*b* = 9.007 (3) Å
*c* = 12.734 (4) Å
α = 107.565 (4)°
β = 94.068 (4)°
γ = 99.092 (4)°
*V* = 908.2 (5) Å^3^
*Z* = 2
Mo *K*α radiation
μ = 0.08 mm^−1^
*T* = 296 K
0.16 × 0.12 × 0.08 mm

#### Data collection

Bruker APEXII CCD diffractometer
Absorption correction: multi-scan (*SADABS*; Bruker, 2005 ▶) *T*_min_ = 0.987, *T*_max_ = 0.993
6434 measured reflections
3159 independent reflections
2612 reflections with *I* > 2σ(*I*)
*R*_int_ = 0.016

#### Refinement

*R*[*F* ^2^ > 2σ(*F* ^2^)] = 0.041
*wR*(*F* ^2^) = 0.117
*S* = 1.06
3159 reflections
240 parameters
H atoms treated by a mixture of independent and constrained refinement
Δρ_max_ = 0.28 e Å^−3^
Δρ_min_ = −0.13 e Å^−3^

### 

Data collection: *APEX2* (Bruker, 2005 ▶); cell refinement: *SAINT* (Bruker, 2005 ▶); data reduction: *SAINT*; program(s) used to solve structure: *SHELXS97* (Sheldrick, 2008 ▶); program(s) used to refine structure: *SHELXL97* (Sheldrick, 2008 ▶); molecular graphics: *SHELXTL* (Sheldrick, 2008 ▶); software used to prepare material for publication: *SHELXTL*.

## Supplementary Material

Crystal structure: contains datablock(s) I, global. DOI: 10.1107/S1600536812019940/rz2747sup1.cif

Structure factors: contains datablock(s) I. DOI: 10.1107/S1600536812019940/rz2747Isup2.hkl

Supplementary material file. DOI: 10.1107/S1600536812019940/rz2747Isup3.cml

Additional supplementary materials:  crystallographic information; 3D view; checkCIF report

## Figures and Tables

**Table 1:** Hydrogen-bond geometry (Å, °)

*D*—H⋯*A*	*D*—H	H⋯*A*	*D*⋯*A*	*D*—H⋯*A*
O1—H1*B*⋯N2	0.94 (3)	2.53 (3)	3.219 (2)	130 (2)
O1—H1*B*⋯N3	0.94 (3)	1.95 (3)	2.790 (2)	148 (2)
C3—H3*A*⋯N2^i^	0.93	2.59	3.390 (3)	145

Symmetry code: (i) 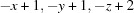.

**Fig. 1.**
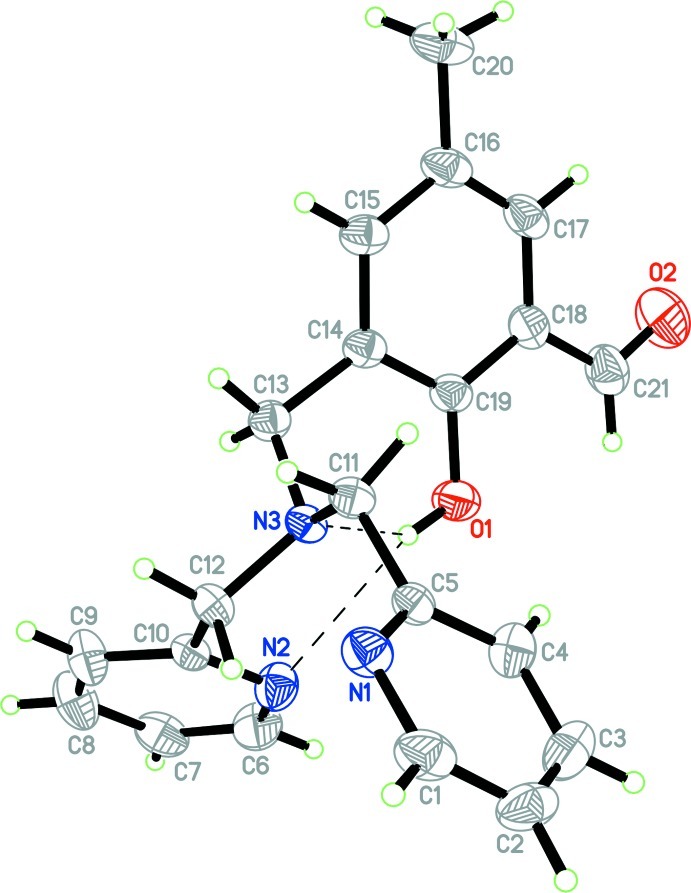
The molecular structure of the title compound showing displacement ellipsoids drawn at the 30% probability level. Intramolecular hydrogen bonds are drawn as dashed lines.

**Fig. 2.**
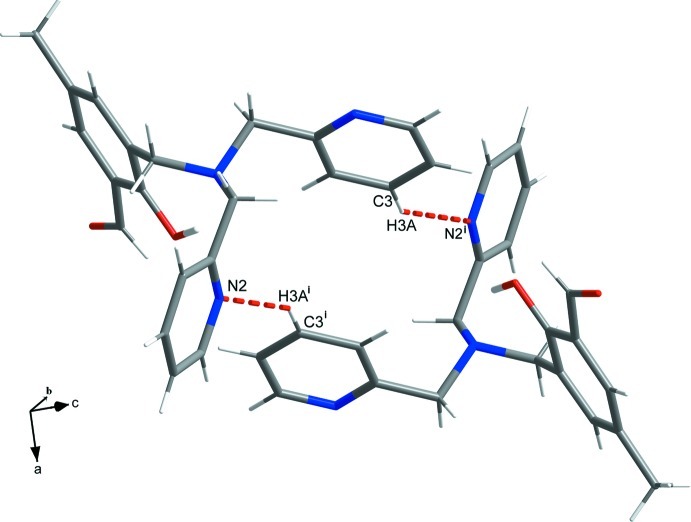
Partial crystal packing of the title compound showing the formation of a dimer through hydrogen bonds (dashed lines). Symmetry code: (i) 1-x, 1-y, 2-z.

## supplementary crystallographic information

### Comment

Unsymmetrical phenolate based "end-off" compartmental ligands which contain two adjacent, but dissimilar coordination sites, are important chelating agents to synthetic model complexes for metalloenzymes such as phosphatase, urease, superoxide dismutase, catalase, tyrosinase, nuclease, *etc* (Lambert *et al.*, 1997; Dubois, Xiang *et al.*, 2003; Dubois, Caspar *et al.*, 2003; Carlsson *et al.*, 2004). The title compound (HL) is a key precursor to the unsymmetrical ligands (Chirakul *et al.*, 2000; Abe *et al.*, 2006). Up to now, different kinds of interesting model complexes with HL and its derivatives have been reported (Lambert *et al.*, 1997; Koval *et al.*, 2003; Koval, Huisman, Stassen, Gamez, Lutz, Spek & Reedijk, 2004; Koval, Huisman, Stassen, Gamez, Lutz, Spek, Pursche *et al.*, 2004; Koval *et al.*, 2007), however, the crystal structure of the HL itself is still unknown to us. Recently, in the synthesis of HL derivatives (Zhu *et al.*, 2007), we unexpectedly obtained single crystals of HL, and its crystal structure is reported herein.

The X-ray analysis of the title compound (Fig. 1) indicated that two pyridyne rings and the substituted phenyl ring lie in a propeller arrangement around the central tertiary amine N3 atom. The dihedral angles between the phenyl ring and two pyridyne rings are 61.0 (3)° and 49.6 (3)°, respectively, while the dihedral angle between two pyridyne rings is 69.7 (3)°. These angles are larger than those found in the ligand L of the related complexes [Cu_2_L(*µ*-NO_3_)(NO_3_)_2_].CH_3_CN, [Cu(HL)Br_2_].0.5H_2_O, [Mn(HL)Cl_2_], (Koval, Huisman, Stassen, Gamez, Lutz, Spek & Reedijk, 2004) and [Co_2_L_2_](ClO_4_)_2_.0.7CH_3_OH, [Co_2_L_2_](BF_4_)_2_.CH_3_OH, [Mn_2_L_2_](BF_4_)_2_.C_4_H_10_O (Koval, Huisman, Stassen, Gamez, Lutz, Spek, Pursche *et al.*, 2004). The dihedral angle between the phenyl ring and the C18/C21/O2 plane through the aldehyde group is 10.5 (3)°. All bond lengths and angles are normal (Bortoluzzi *et al.*, 2007). An intramolecular bifurcate O—H···N hydrogen bond (Table 1) stabilizes the molecular conformation. In the crystal structure (Fig. 2), centrosymmetrically related molecules are linked by pairs of C—H···N hydrogen bonds into dimers.

### Experimental

The title compound was synthesized following the method reported by Lambert *et al.* (1997) and Koval *et al.* (2003). Diffraction quality crystals were obtained by slow evaporation of an acetone solution.

### Refinement

The hydroxy H atom was located in a difference Fourier map and refined freely. All other H atoms were positioned geometrically and allowed to ride on their parent atoms with C—H = 0.93–0.97 Å, and with *U*_iso_(H) = 1.2 *U*_eq_(C) or 1.5 *U*_eq_(C) for methyl H atoms.

### Crystal data

**Table:**

C_21_H_21_N_3_O_2_	*Z* = 2
*M**_r_* = 347.41	*F*(000) = 368
Triclinic, *P*1	*D*_x_ = 1.270 Mg m^−^^3^
Hall symbol: -P 1	Mo *K*α radiation, λ = 0.71073 Å
*a* = 8.479 (3) Å	Cell parameters from 285 reflections
*b* = 9.007 (3) Å	θ = 1.7–26.9°
*c* = 12.734 (4) Å	µ = 0.08 mm^−^^1^
α = 107.565 (4)°	*T* = 296 K
β = 94.068 (4)°	Prism, colourless
γ = 99.092 (4)°	0.16 × 0.12 × 0.08 mm
*V* = 908.2 (5) Å^3^	

### Data collection

**Table:**

Bruker APEXII CCD diffractometer	3159 independent reflections
Radiation source: fine-focus sealed tube	2612 reflections with *I* > 2σ(*I*)
Graphite monochromator	*R*_int_ = 0.016
ω scans	θ_max_ = 25.0°, θ_min_ = 1.7°
Absorption correction: multi-scan (*SADABS*; Bruker, 2005)	*h* = −10→9
*T*_min_ = 0.987, *T*_max_ = 0.993	*k* = −10→9
6434 measured reflections	*l* = −15→15

### Refinement

**Table:**

Refinement on *F*^2^	Secondary atom site location: difference Fourier map
Least-squares matrix: full	Hydrogen site location: inferred from neighbouring sites
*R*[*F*^2^ > 2σ(*F*^2^)] = 0.041	H atoms treated by a mixture of independent and constrained refinement
*wR*(*F*^2^) = 0.117	*w* = 1/[σ^2^(*F*_o_^2^) + (0.0586*P*)^2^ + 0.1506*P*] where *P* = (*F*_o_^2^ + 2*F*_c_^2^)/3
*S* = 1.06	(Δ/σ)_max_ = 0.002
3159 reflections	Δρ_max_ = 0.28 e Å^−^^3^
240 parameters	Δρ_min_ = −0.13 e Å^−^^3^
0 restraints	Extinction correction: *SHELXL97* (Sheldrick, 2008), Fc^*^=kFc[1+0.001xFc^2^λ^3^/sin(2θ)]^-1/4^
Primary atom site location: structure-invariant direct methods	Extinction coefficient: 0.015 (4)

### Special details

**Table:**

Geometry. All e.s.d.'s (except the e.s.d. in the dihedral angle between two l.s. planes) are estimated using the full covariance matrix. The cell e.s.d.'s are taken into account individually in the estimation of e.s.d.'s in distances, angles and torsion angles; correlations between e.s.d.'s in cell parameters are only used when they are defined by crystal symmetry. An approximate (isotropic) treatment of cell e.s.d.'s is used for estimating e.s.d.'s involving l.s. planes.
Refinement. Refinement of *F*^2^ against ALL reflections. The weighted *R*-factor *wR* and goodness of fit *S* are based on *F*^2^, conventional *R*-factors *R* are based on *F*, with *F* set to zero for negative *F*^2^. The threshold expression of *F*^2^ > σ(*F*^2^) is used only for calculating *R*-factors(gt) *etc*. and is not relevant to the choice of reflections for refinement. *R*-factors based on *F*^2^ are statistically about twice as large as those based on *F*, and *R*- factors based on ALL data will be even larger.

### Fractional atomic coordinates and isotropic or equivalent isotropic displacement parameters (Å^2^)

**Table:**

	*x*	*y*	*z*	*U*_iso_*/*U*_eq_	
O1	0.30347 (14)	0.24261 (15)	0.70069 (11)	0.0612 (4)	
O2	0.2793 (2)	0.4303 (2)	0.45307 (13)	0.0963 (5)	
N1	0.08654 (17)	0.36504 (16)	1.11217 (10)	0.0500 (3)	
N2	0.44036 (16)	0.04369 (16)	0.84649 (12)	0.0556 (4)	
N3	0.13178 (14)	0.13408 (13)	0.85047 (9)	0.0373 (3)	
C1	0.1725 (2)	0.4744 (2)	1.20326 (14)	0.0619 (5)	
H1A	0.1422	0.4755	1.2722	0.074*	
C2	0.3018 (2)	0.5841 (2)	1.20004 (17)	0.0672 (5)	
H2A	0.3586	0.6568	1.2652	0.081*	
C3	0.3457 (2)	0.5845 (2)	1.09924 (19)	0.0677 (5)	
H3A	0.4331	0.6578	1.0944	0.081*	
C4	0.2591 (2)	0.47535 (19)	1.00495 (15)	0.0544 (4)	
H4A	0.2865	0.4747	0.9354	0.065*	
C5	0.13093 (17)	0.36626 (16)	1.01410 (12)	0.0386 (3)	
C6	0.5620 (2)	−0.0253 (2)	0.80858 (17)	0.0649 (5)	
H6A	0.6501	0.0376	0.7938	0.078*	
C7	0.5662 (2)	−0.1817 (2)	0.79005 (16)	0.0644 (5)	
H7A	0.6543	−0.2242	0.7636	0.077*	
C8	0.4367 (3)	−0.2741 (2)	0.81160 (18)	0.0719 (6)	
H8A	0.4350	−0.3813	0.8001	0.086*	
C9	0.3086 (2)	−0.2069 (2)	0.85051 (16)	0.0599 (5)	
H9A	0.2190	−0.2683	0.8648	0.072*	
C10	0.31508 (17)	−0.04723 (17)	0.86807 (12)	0.0400 (3)	
C11	0.03455 (17)	0.24405 (18)	0.91278 (12)	0.0414 (3)	
H11A	−0.0550	0.1841	0.9350	0.050*	
H11B	−0.0093	0.2971	0.8647	0.050*	
C12	0.18157 (18)	0.03443 (18)	0.91383 (12)	0.0433 (4)	
H12A	0.0896	−0.0450	0.9134	0.052*	
H12B	0.2170	0.0996	0.9903	0.052*	
C13	0.04151 (18)	0.03472 (17)	0.74274 (12)	0.0426 (4)	
H13A	−0.0639	−0.0127	0.7545	0.051*	
H13B	0.0976	−0.0504	0.7096	0.051*	
C14	0.02146 (18)	0.12748 (17)	0.66384 (11)	0.0417 (4)	
C15	−0.1264 (2)	0.11424 (19)	0.60476 (12)	0.0488 (4)	
H15A	−0.2157	0.0511	0.6179	0.059*	
C16	−0.1472 (2)	0.1916 (2)	0.52628 (13)	0.0540 (4)	
C17	−0.0125 (2)	0.28009 (19)	0.50600 (13)	0.0548 (4)	
H17A	−0.0229	0.3306	0.4527	0.066*	
C18	0.1394 (2)	0.29671 (18)	0.56266 (12)	0.0496 (4)	
C19	0.15547 (19)	0.22339 (18)	0.64412 (12)	0.0447 (4)	
C20	−0.3128 (3)	0.1731 (3)	0.46591 (17)	0.0776 (6)	
H20A	−0.3064	0.2332	0.4151	0.116*	
H20B	−0.3843	0.2111	0.5187	0.116*	
H20C	−0.3529	0.0631	0.4256	0.116*	
C21	0.2815 (3)	0.3831 (2)	0.53231 (16)	0.0656 (5)	
H21A	0.3792	0.4027	0.5768	0.079*	
H1B	0.286 (3)	0.207 (3)	0.762 (2)	0.088 (7)*	

### Atomic displacement parameters (Å^2^)

**Table:**

	*U*^11^	*U*^22^	*U*^33^	*U*^12^	*U*^13^	*U*^23^
O1	0.0500 (7)	0.0767 (9)	0.0618 (8)	0.0016 (6)	−0.0033 (6)	0.0369 (7)
O2	0.1206 (13)	0.1079 (12)	0.0805 (10)	0.0155 (10)	0.0190 (9)	0.0608 (10)
N1	0.0583 (8)	0.0525 (8)	0.0402 (7)	0.0139 (6)	0.0080 (6)	0.0143 (6)
N2	0.0447 (8)	0.0503 (8)	0.0726 (9)	0.0084 (6)	0.0122 (7)	0.0196 (7)
N3	0.0409 (7)	0.0406 (7)	0.0325 (6)	0.0132 (5)	0.0043 (5)	0.0118 (5)
C1	0.0779 (13)	0.0663 (12)	0.0402 (9)	0.0278 (10)	0.0011 (8)	0.0092 (8)
C2	0.0591 (12)	0.0548 (11)	0.0687 (13)	0.0207 (9)	−0.0150 (9)	−0.0082 (9)
C3	0.0483 (10)	0.0476 (10)	0.0942 (15)	0.0050 (8)	0.0077 (10)	0.0055 (10)
C4	0.0536 (10)	0.0487 (9)	0.0611 (10)	0.0094 (8)	0.0163 (8)	0.0157 (8)
C5	0.0392 (8)	0.0392 (8)	0.0411 (8)	0.0155 (6)	0.0067 (6)	0.0139 (6)
C6	0.0413 (9)	0.0742 (12)	0.0825 (13)	0.0128 (9)	0.0133 (9)	0.0277 (10)
C7	0.0553 (11)	0.0843 (14)	0.0662 (11)	0.0374 (10)	0.0134 (9)	0.0288 (10)
C8	0.0888 (14)	0.0615 (11)	0.0865 (14)	0.0411 (11)	0.0302 (12)	0.0363 (11)
C9	0.0681 (12)	0.0514 (10)	0.0752 (12)	0.0212 (9)	0.0267 (9)	0.0330 (9)
C10	0.0412 (8)	0.0450 (8)	0.0369 (7)	0.0104 (6)	0.0020 (6)	0.0172 (6)
C11	0.0396 (8)	0.0472 (8)	0.0389 (8)	0.0139 (6)	0.0059 (6)	0.0126 (6)
C12	0.0486 (9)	0.0467 (8)	0.0413 (8)	0.0146 (7)	0.0100 (7)	0.0199 (7)
C13	0.0441 (8)	0.0422 (8)	0.0390 (8)	0.0079 (6)	0.0039 (6)	0.0094 (6)
C14	0.0466 (9)	0.0431 (8)	0.0316 (7)	0.0126 (7)	0.0018 (6)	0.0050 (6)
C15	0.0493 (9)	0.0521 (9)	0.0389 (8)	0.0147 (7)	0.0011 (7)	0.0042 (7)
C16	0.0635 (11)	0.0561 (10)	0.0374 (8)	0.0255 (8)	−0.0058 (7)	0.0032 (7)
C17	0.0792 (13)	0.0498 (9)	0.0364 (8)	0.0240 (9)	−0.0012 (8)	0.0114 (7)
C18	0.0666 (11)	0.0433 (8)	0.0389 (8)	0.0148 (8)	0.0045 (7)	0.0112 (7)
C19	0.0494 (9)	0.0458 (8)	0.0369 (8)	0.0111 (7)	0.0004 (6)	0.0102 (7)
C20	0.0765 (14)	0.0919 (15)	0.0607 (12)	0.0335 (12)	−0.0152 (10)	0.0153 (11)
C21	0.0856 (14)	0.0608 (11)	0.0568 (11)	0.0146 (10)	0.0119 (10)	0.0272 (9)

### Geometric parameters (Å, º)

**Table:**

O1—C19	1.3607 (19)	C9—C10	1.378 (2)
O1—H1B	0.94 (2)	C9—H9A	0.9300
O2—C21	1.207 (2)	C10—C12	1.503 (2)
N1—C5	1.3324 (19)	C11—H11A	0.9700
N1—C1	1.346 (2)	C11—H11B	0.9700
N2—C6	1.332 (2)	C12—H12A	0.9700
N2—C10	1.333 (2)	C12—H12B	0.9700
N3—C12	1.4640 (18)	C13—C14	1.505 (2)
N3—C13	1.4704 (19)	C13—H13A	0.9700
N3—C11	1.4719 (18)	C13—H13B	0.9700
C1—C2	1.367 (3)	C14—C15	1.384 (2)
C1—H1A	0.9300	C14—C19	1.401 (2)
C2—C3	1.363 (3)	C15—C16	1.396 (2)
C2—H2A	0.9300	C15—H15A	0.9300
C3—C4	1.372 (3)	C16—C17	1.372 (3)
C3—H3A	0.9300	C16—C20	1.512 (2)
C4—C5	1.382 (2)	C17—C18	1.394 (2)
C4—H4A	0.9300	C17—H17A	0.9300
C5—C11	1.501 (2)	C18—C19	1.397 (2)
C6—C7	1.364 (3)	C18—C21	1.469 (3)
C6—H6A	0.9300	C20—H20A	0.9600
C7—C8	1.365 (3)	C20—H20B	0.9600
C7—H7A	0.9300	C20—H20C	0.9600
C8—C9	1.376 (3)	C21—H21A	0.9300
C8—H8A	0.9300		
			
C19—O1—H1B	106.1 (14)	H11A—C11—H11B	107.9
C5—N1—C1	117.31 (15)	N3—C12—C10	112.76 (11)
C6—N2—C10	117.32 (15)	N3—C12—H12A	109.0
C12—N3—C13	110.23 (11)	C10—C12—H12A	109.0
C12—N3—C11	111.29 (11)	N3—C12—H12B	109.0
C13—N3—C11	110.37 (11)	C10—C12—H12B	109.0
N1—C1—C2	123.62 (17)	H12A—C12—H12B	107.8
N1—C1—H1A	118.2	N3—C13—C14	112.35 (12)
C2—C1—H1A	118.2	N3—C13—H13A	109.1
C3—C2—C1	118.53 (17)	C14—C13—H13A	109.1
C3—C2—H2A	120.7	N3—C13—H13B	109.1
C1—C2—H2A	120.7	C14—C13—H13B	109.1
C2—C3—C4	119.06 (18)	H13A—C13—H13B	107.9
C2—C3—H3A	120.5	C15—C14—C19	118.35 (14)
C4—C3—H3A	120.5	C15—C14—C13	121.45 (14)
C3—C4—C5	119.49 (17)	C19—C14—C13	120.11 (13)
C3—C4—H4A	120.3	C14—C15—C16	122.85 (16)
C5—C4—H4A	120.3	C14—C15—H15A	118.6
N1—C5—C4	121.98 (14)	C16—C15—H15A	118.6
N1—C5—C11	117.07 (13)	C17—C16—C15	117.36 (15)
C4—C5—C11	120.95 (13)	C17—C16—C20	122.75 (17)
N2—C6—C7	124.44 (17)	C15—C16—C20	119.87 (18)
N2—C6—H6A	117.8	C16—C17—C18	122.13 (15)
C7—C6—H6A	117.8	C16—C17—H17A	118.9
C6—C7—C8	117.75 (16)	C18—C17—H17A	118.9
C6—C7—H7A	121.1	C17—C18—C19	119.25 (16)
C8—C7—H7A	121.1	C17—C18—C21	120.01 (15)
C7—C8—C9	119.36 (17)	C19—C18—C21	120.66 (16)
C7—C8—H8A	120.3	O1—C19—C18	118.99 (15)
C9—C8—H8A	120.3	O1—C19—C14	121.01 (13)
C8—C9—C10	119.11 (17)	C18—C19—C14	119.96 (15)
C8—C9—H9A	120.4	C16—C20—H20A	109.5
C10—C9—H9A	120.4	C16—C20—H20B	109.5
N2—C10—C9	122.01 (14)	H20A—C20—H20B	109.5
N2—C10—C12	116.25 (13)	C16—C20—H20C	109.5
C9—C10—C12	121.74 (14)	H20A—C20—H20C	109.5
N3—C11—C5	112.23 (12)	H20B—C20—H20C	109.5
N3—C11—H11A	109.2	O2—C21—C18	124.0 (2)
C5—C11—H11A	109.2	O2—C21—H21A	118.0
N3—C11—H11B	109.2	C18—C21—H21A	118.0
C5—C11—H11B	109.2		

### Hydrogen-bond geometry (Å, º)

**Table:**

*D*—H···*A*	*D*—H	H···*A*	*D*···*A*	*D*—H···*A*
O1—H1*B*···N2	0.94 (3)	2.53 (3)	3.219 (2)	130 (2)
O1—H1*B*···N3	0.94 (3)	1.95 (3)	2.790 (2)	148 (2)
C3—H3*A*···N2^i^	0.93	2.59	3.390 (3)	145

Symmetry code: (i) −*x*+1, −*y*+1, −*z*+2.
